# Heterochrony and developmental modularity of cranial osteogenesis in lipotyphlan mammals

**DOI:** 10.1186/2041-9139-2-21

**Published:** 2011-11-01

**Authors:** Daisuke Koyabu, Hideki Endo, Christian Mitgutsch, Gen Suwa, Kenneth C Catania, Christoph PE Zollikofer, Sen-ichi Oda, Kazuhiko Koyasu, Motokazu Ando, Marcelo R Sánchez-Villagra

**Affiliations:** 1 Paläontologisches Institut und Museum, Universität Zürich, Karl Schmid-Strasse 4, CH-8006 Zürich, Switzerland; 2 The University Museum, The University of Tokyo, Hongo 7-3-1, Bunkyo-ku, 113-0033 Tokyo, Japan; 3 Department of Biological Sciences, Vanderbilt University, VU Station B, Box 35-1634, Nashville, USA; 4 Anthropologisches Institut, Universität Zürich, Winterthurerstrasse 190, CH-8057 Zurich, Switzerland; 5 Department of Zoology, Okayama University of Science, Ridaichou, Kita-ku, 700-0005 Okayama, Japan; 6 The Second Department of Anatomy, School of Dentistry, Aichi-Gakuin University, Kusumotochou 1-100, 464-8650 Nagoya, Japan; 7 Faculty of Agriculture, Tokyo University of Agriculture, Funako 1737, 243-0034 Atsugi, Japan

**Keywords:** skull, heterochrony, Eulipotyphla, embryology, ossification, integration, phylogeny, micro CT

## Abstract

**Background:**

Here we provide the most comprehensive study to date on the cranial ossification sequence in Lipotyphla, the group which includes shrews, moles and hedgehogs. This unique group, which encapsulates diverse ecological modes, such as terrestrial, subterranean, and aquatic lifestyles, is used to examine the evolutionary lability of cranial osteogenesis and to investigate the modularity of development.

**Results:**

An acceleration of developmental timing of the vomeronasal complex has occurred in the common ancestor of moles. However, ossification of the nasal bone has shifted late in the more terrestrial shrew mole. Among the lipotyphlans, sequence heterochrony shows no significant association with modules derived from developmental origins (that is, neural crest cells vs. mesoderm derived parts) or with those derived from ossification modes (that is, dermal vs. endochondral ossification).

**Conclusions:**

The drastic acceleration of vomeronasal development in moles is most likely coupled with the increased importance of the rostrum for digging and its use as a specialized tactile surface, both fossorial adaptations. The late development of the nasal in shrew moles, a condition also displayed by hedgehogs and shrews, is suggested to be the result of an ecological reversal to terrestrial lifestyle and reduced functional importance of the rostrum. As an overall pattern in lipotyphlans, our results reject the hypothesis that ossification sequence heterochrony occurs in modular fashion when considering the developmental patterns of the skull. We suggest that shifts in the cranial ossification sequence are not evolutionarily constrained by developmental origins or mode of ossification.

## Background

The mammalian skull is one of the most extensively studied anatomical systems among vertebrate structures. One of the major aspects of morphological evolution in the mammalian skull is heterochrony, shifts in the timing and rate of development. The more classic heterochronic studies have focused on quantifying changes in size and shape, whereas the study of 'sequence' heterochrony incorporates changes in the timing of discrete developmental events with many advantages in comparative studies [1-4]. One set of events that has received much attention in recent years is the onset of ossification of individual bones [[5] and references therein]. Sánchez-Villagra *et al. *[6], presenting the most comprehensive study of sequence heterochrony in the mammalian skull to date, analyzed the ossification sequence of 17 bone elements of the skull among 7 marsupial and 13 species of boreoeutherians (the placental mammals, excluding afrotherians and xenarthrans [7]). A few heterochronies were found to diagnose mammals, marsupials or placentals, but the relative timing of cranial ossification patterns is largely conserved among mammal evolution. Although similarly conserved developmental patterns have been reported for Rodentia [8], further investigation of unexamined species and more phylogenetically inclusive studies are needed to test whether this conservatism is a general pattern of mammals.

Recently, the connection of heterochrony to modularity, another central aspect in the evolution of development, has attracted much attention in vertebrates in general [for example, [9-16]]. Modularity, which is considered to be one of the key concepts to bridge evolutionary biology and developmental biology [17-21], refers to the autonomy of groups of events or structures, as well as the strong associations of developmental events or morphological structures [22]. Independence among anatomical structures is thought to permit unrelated parts to vary and/or evolve separately, while the integration within smaller units maintains functionally necessary relationships among traits [23]. From studies on the genetic, developmental and functional modules across vertebrates, it has been implied that developmental modularity may provide insights into processes of morphological evolution [24-34]. Most studies have focused on the physical relationships among functionally- or developmentally-related structures, and there are only a few studies relating developmental timing to the concept of modularity [9-11,35].

Heterochrony is only considered possible in a modular phenotype, in which some parts are autonomous developmentally from others [10,15,36-38]. It has been suggested that heterochronic change in ossification events of the tetrapod skull occurs among different developmental modules, while maintaining the relative timing of developmental events in each module [11]. Schoch [11] suggested that sets of cranial bones belonging to the same developmental modules could shift ossification timing in unison, although this proposition was not statistically tested. Using the analytical method developed by Poe [39], a study on several therian mammals found that postcranial modules show significant developmental integration of the entire appendicular skeleton in boreoeutherian placentals [10]. However, cranial phenotypic variational modules showed no significant conservation of developmental timing for all sister group comparisons, except within a clade consisting of a mole and a shrew, which displayed integrated shifts of ossification sequence in the facial module [10]. While ossification timing of some skeletal parts was implied to reflect evolutionary modularity in some taxa, it remains unclear whether, and to what degree, heterochrony involves modularity. Although the onset of the ossification sequence of cranial parts in the facial module was indicated to shift together within the mole-shrew clade (*Talpa europaea *and *Cryptotis parva*), a detailed and more inclusive study of the diverse group to which these animals belong is needed to strictly test the hypothesis relating sequence heterochrony to modularity. In addition, although a significant relationship between cranial heterochrony and modularity was not found in most taxa examined [10], it is still possible that shifts in the cranial ossification sequence of some taxa are less constrained by developmental modularity, whereas those of other taxa may be strongly constrained by modular pattern. Furthermore, because only the relationship between patterns of sequence heterochrony and modularity of phenotypic variation was compared by Goswami [10], it is yet unclear whether developmental modularity of sequence heterochrony is associated with modules derived from developmental origins (that is, neural crest cells vs. mesoderm derived parts) and/or with those derived from developmental modes (that is, dermal vs. endochondral ossification). The body parts that share developmental origins and functions are suggested to behave as evolutionary modules at various levels [23,26,40]. Therefore, we hypothesize that sequence heterochrony is more likely to occur among different developmental modules, while maintaining the relative timing of developmental events within a single module.

Here we provide the most comprehensive study to date on the ossification sequence of cranial elements in Lipotyphla, the group which includes shrews (Soricidae), moles and 'shrew moles' (Talpidae), hedgehogs (Erinaceidae), and Solenodons (Solenodontidae) [41]. With an extensive series of embryonic specimens from a variety of taxa, we present data on cranial ossification sequences for two species of terrestrial hedgehogs (*Erinaceus europaeus *and *E. amurensis*), three species of subterranean mole (*Mogera wogura*, *Condylura cristata*, and *Scapanus orarius*), one terrestrial shrew mole (*Urotrichus talpoides*), one terrestrial shrew (*Suncus murinus*), and an aquatic water shrew (*Chimarrogale platycephala*). In addition, the resolution of sequence data for two moles (*Talpa europaea *and *T. occidentalis*) and one shrew species (*Cryptotis parva*) is much improved compared with the data presented in previous literature [6,42,43]. Using this unique group that encapsulates terrestrial, subterranean and aquatic lifestyles, we examine the evolutionary lability and conservatism of cranial osteogenesis and test the hypothesis that skeletal elements belonging to the same module display coordinated shifts of ossification timing while maintaining the relative sequence within the module.

## Materials and methods

### Data acquisition

**Figure 1:**
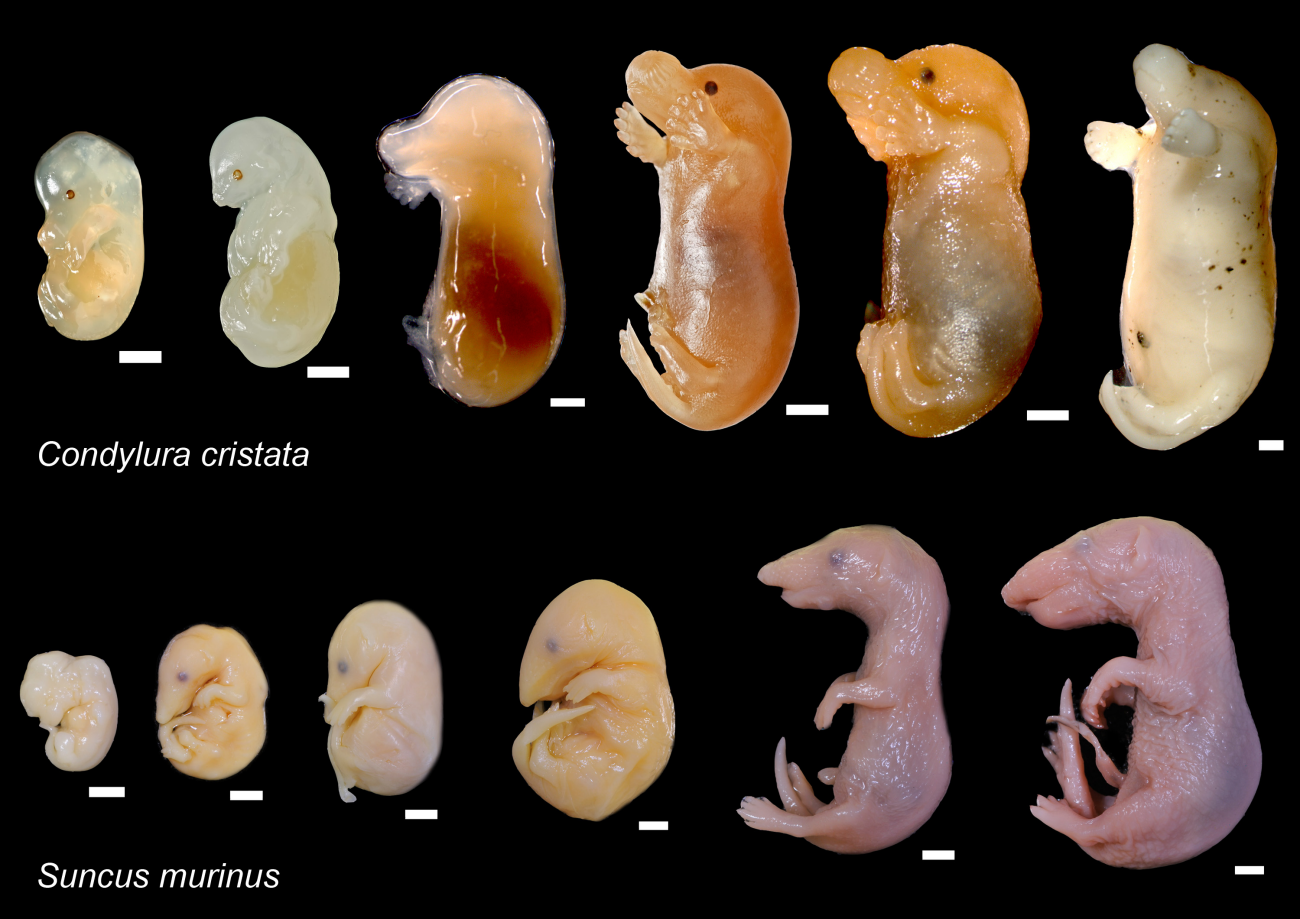
**Ontogenetic series prepared for this study**. Upper: *Condylura cristata*. Lower: *Suncus murinus*. Scale bar, 2 mm.

**Figure 2:**
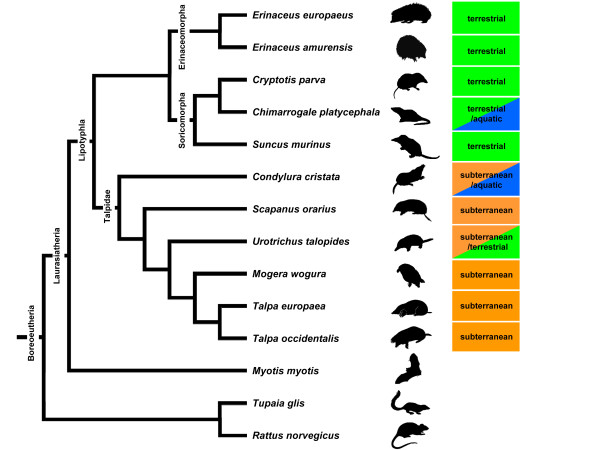
**Phylogenetic relationships among the lipotyphlan species and outgroups included in this study**. The phylogenetic framework on which the data were examined is a composite of several sources [47,51,52].

Ossification sequence data of 22 cranial elements in 11 lipotyphlan species were sampled from 238 embryonic specimens held in the collections at the Paleontological Institute and Museum of University of Zürich, Kyoto University Museum, Botanical Gardens Museum of Hokkaido University, Aichigakuin University Dental Science Museum, and Wildlife Laboratory at Tokyo University of Agriculture (Figure 1, Table 1). The jugal and ethmoid were excluded from the analysis because these bones were exceptionally small and difficult to indentify. Data for three boreoeutherian outgroup species, including the greater mouse-eared bat (*Myotis myotis*), the common treeshrew (*Tupaia glis*), and the Norway rat (*Rattus norvegicus*), were obtained from the literature [44-46]. Figure 2 is a composite consensus phylogenetic tree of the studied species based on molecular and morphological analyses [47-52].

**Table 1:** Species names, specimen, and stage number

Species name	Specimen numbers/stages	References
*Erinaceus europaeus*	10/7	this study
*Erinaceus amurensis*	21/9	this study
*Cryptotis parva*	29/13	this study
*Chimarrogale platycephala*	30/8	this study
*Suncus murinus*	49/11	this study
*Condylura cristata*	10/9	this study
*Scapanus orarius*	14/7	this study
*Urotrichus talpoides*	13/8	this study
*Mogera wogura*	16/8	this study
*Talpa europaea*	11/7	this study
*Talpa occidentalis*	35/10	this study
*Myotis myotis*	26/12	[28]
*Tupaia glis*	26/11	[30]
*Rattus norvegicus*	n.a./7	[29]

**Figure 3:**
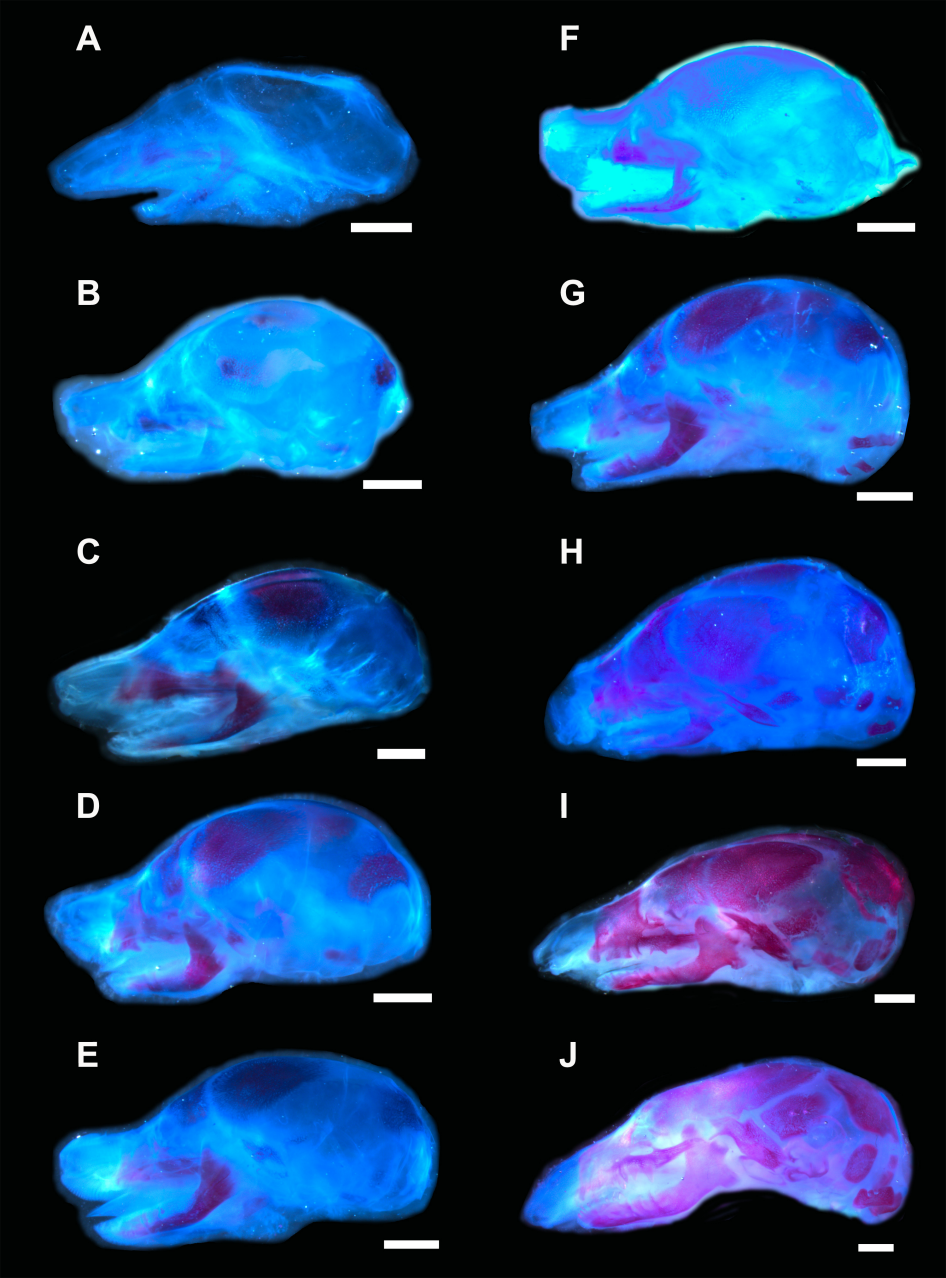
**Cleared and stained specimens of *Talpa occidentalis***. Calcified structures are stained in red, cartilages in blue, and connective tissue in light blue. Scale bar, 2 mm.

**Figure 4:**
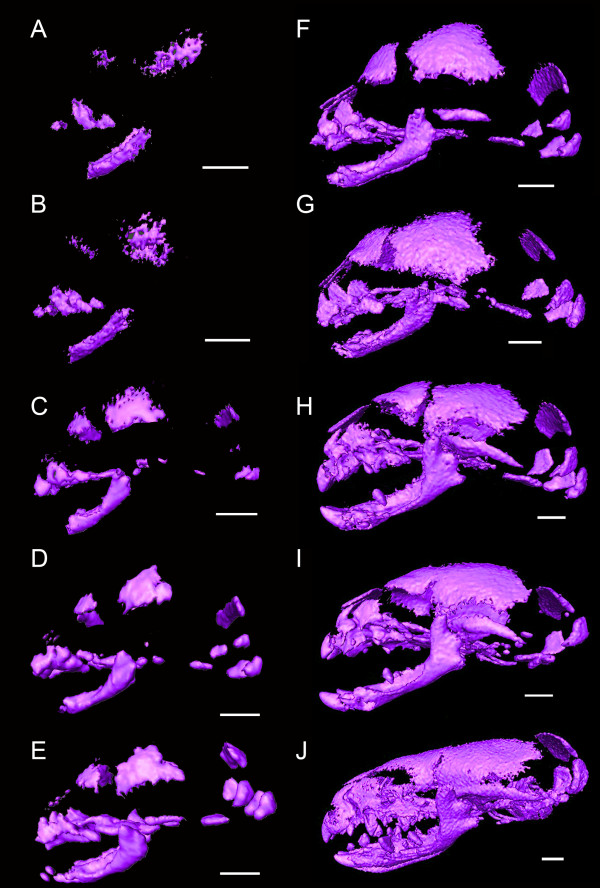
**Three-dimensional reconstructions of μCT scans of *Suncus murinus***. Scale bar, 1 mm.

We employed an enzymatic clearing and staining method and a high-resolution tomography (μCT) technique for detecting ossification. Some specimens were cleared and stained by a modified method of a standard enzymatic procedure [43], and the earliest appearance of ossifications was recorded based on uptake of alizarin red (Figure 3) [6]. As other species were historical museum specimens, the appearance of bones was also assessed noninvasively by acquiring shadow images taken by μCT at the University Museum, University of Tokyo (TXS225-ACTIS, TESCO, Tokyo) and at the Anthropological Institute, University of Zurich (μCT80, Scano Medical, Bassersdorf, Switzerland). Three dimensional visualization and analysis of shadow images were conducted in Avizo 6.1 (Visualization Sciences Group, Burlington, MA, USA) (Figure 4). Since using different visualization techniques to obtain ossification sequence is reported not to represent a confounding issue and that differences in detection thresholds do not yield erroneous sequences [53,54], we consider minimum error will be involved with employing both clear staining and μCT methods.

### Analysis of variation in ossification sequence

To examine the rank variation in sequence of a particular ossification event, we scaled the rank of each ossification event as:

$$\left(r-1\right)∕\left(r_{\max}-1\right)$$

in which *r *is the absolute rank of a given ossification event, and *r*_max _is the total number of ranks for each species. Therefore, the relative ranks of each species are distributed between 0 and 1. This allowed us to remove the differences of maximum rank between species resulting from differing levels of sampling resolution between species. A similar approach as standardizing the absolute rank *r *by the maximum number of ranks (*r*_max_) has been applied in previous sequence heterochrony studies [for example, [6,8,53]]. As the ranks are distributed between 1/*r*_max _and 1 with this method, the relative ranks of the earliest bone to ossify can vary, depending on *r*_max_. However, the method used here circumvents this problem because the relative ranks of the earliest event will always be scaled to zero. Nevertheless, some noise remains because species with higher *r*_max _have a lower influence on the variance [53]. The range in rank variation across species was assessed to examine the variability of a particular element in the ossification sequence. The frequency distribution of ranks was also calculated to examine the distribution of ossification events within the rank sequence. In order to assess the variability of ossification timing between modules, rank variation between hypothetical modules was also compared.

### Event pairing and PARSIMOV analysis

To identify heterochronies within the ossification sequence, the timing of each ossification event of 22 bones was compared with every other ossification event, that is, the ossification rank of one bone was compared with that of another bone within the species (Table 2). This resulted in 231 event pairs for each species, which were treated as 'characters' [55]. Three character states, before (score 0), simultaneous (score 1), or after (score 2), were given respectively to reflect the relative timing of one ossification event relative to another. For example, if the premaxilla is earlier than the nasal in a certain species, then this event pair is scored as 0. In this way, differences in sequence resolution can be overcome and event pairs can be compared among species. Simultaneous events are usually the result of low resolution of sampling, because the onset of ossification of two bones is unlikely to occur exactly at the same time [6,56]. After constructing event pairs, the character state at each node of the phylogeny can be inferred by a parsimonious approach (but see [57]). Parsimov [55] was employed to map event pairs onto the given phylogeny (Figure 2) and to analyze the evolutionary change of developmental timing. Outgroup species (*M. myotis*, *T. glis*, and *R. norvegicus*) were used to polarize the characters. This parsimony-based phylogenetic method investigates all possible sets of event timing changes on each branch of the phylogeny. Then it computes the minimal number of heterochronic events that account for every event-pair change and yields a consensus that contains all hypotheses of movement that must necessarily form part of any equally parsimonious solution to the observed event-pair changes [55]. This analysis provides a conservative estimate of change compared to the simple mapping method or the more subjective cracking method [6,58]. As recommended by Jeffery *et al. *[55], optimizations were performed using both ACCTRAN and DELTRAN options, and the consensus of the two was accepted as the most conservative estimate of heterochronic shifts. It should be noted that Parsimov is a highly conservative method and that the consensus of ACCTRAN and DELTRAN is an estimate of minimal heterochronic changes [59].

**Table 2:** Cranial events ranked according to relative timing of onset of ossification

	E. europaeus	E. amurensis	Cr. parva	Ch. platycephala	Su. murinus	Co. cristata	Sc. orarius	U. talpoides	M. wogura	T. europaea	T. occidentalis	Myotis	Tupaia	Rattus
Premaxilla	1	1	1	2	1	1	1	1	1	1	1	2	2	2
Maxilla	1	1	1	2	1	1	1	1	1	1	1	1	2	1
Palatine	3	3	2	2	3	1	1	1	2	1	1	6	3	2
Dentary	1	1	1	1	1	1	1	1	1	1	1	1	1	1
Frontal	1	2	2	2	2	1	1	1	2	1	2	2	4	1
Parietal	4	1	3	2	1	1	1	1	1	1	1	2	4	2
Squamosal	4	3	5	2	4	2	2	2	2	2	3	2	4	2
Basioccipital	5	3	6	3	3	1	5	3	3	2	1	8	6	2
Nasal	4	3	4	2	5	1	1	6	2	2	2	7	7	3
Pterygoid	1	3	3	2	4	1	1	1	2	2	4	3	5	2
Exoccipital	5	3	6	2	4	1	2	4	4	3	1	10	7	2
Basisphenoid	4	3	8	5	6	4	3	5	7	4	6	9	9	4
Lacrimal	6	5	6	6	5	6	1	8	4	4	3	?	5	3
Alisphenoid	5	3	9	6	8	8	7	8	8	7	8	9	7	4
Orbitosphenoid	5	4	10	6	9	7	6	7	6	6	7	12	9	7
Petrosal	7	8	11	7	10	9	7	8	8	7	9	11	11	6
Mastoid	7	9	12	7	11	5	5	6	5	4	3	11	11	7
Vomer	2	3	2	2	5	1	1	1	2	1	1	8	5	2
Presphenoid	7	9	13	8	11	9	7	8	8	7	10	?	10	6
Supraoccipital	5	3	5	2	3	1	2	5	2	2	1	9	8	5
Ectotympanic	6	6	6	4	7	3	2	8	6	5	5	5	6	3
Goniale	6	7	7	6	8	6	4	8	2	5	7	4	6	?

Data for three boreoeutherian outgroup species, the greater mouse-eared bat (*Myotis myotis*), the common treeshrew (*Tupaia glis*), and the Norway rat (*Rattus norvegicus*) were compiled from the literature [44-46]. Simultaneous events are usually the result of low resolution of sampling, since the onset of ossification of two bones is unlikely to occur exactly at the same time. Therefore, the resolutions for oral bones in *Co. cristata*, *Sc. Orarius*, *U. talpoides*, and *T. europaea *are limited in this study.

Although event-pair data are unsuitable for phylogenetic analysis due to non-independence of event pairs [60], and although the main objective of this study is not to infer phylogenetic relationships among lipotyphlans, parsimony analysis can be applied to explore the phylogenetic signal within the data. A parsimony analysis of event-pair data was conducted using PAUP∗ version 4.0b10 [61].

### Modularity analysis

Using the data on the cranial ossification sequence, we tested whether the cranial elements which belong to the same hypothetical module exhibit coordinated shifts in ossification timing [39]. To test for coordinated shifts in ossification timing, theoretical developmental and phenotypic variational modules were first constructed. Theoretical modules are composed of sets of elements that are predicted to exhibit coordinated timing of the first ossification. In this study, three independent hypothetical divisions were tested. Cranial elements were divided into two developmental origin modules, the mesoderm module and neural crest cell module, according to their reported developmental origin in the mouse [62]. Alternatively, cranial elements were classified into another independent developmental division according to the mode of ossification, that is, the endochondral bone module and dermal bone module [63]. Phenotypic variational module division was based on the morphometric analyses, which have identified five sub-divisions in the adult mammalian skull: oral, zygomatic, nasal, cranial base, and cranial vault [34,64,65]. Hypothetical module associations of bone elements used in the analysis are listed in Table 3.

**Table 3:** Hypothetical module associations of bone elements

	Developmental origin module		Ossification mode module		Phenotypic module				
			
	Neural crest cells	Mesoderm	Endochondral	Dermal	Oral	Nasal	Zygomatic	Vault	Basicranium
Premaxilla	◯			◯	◯	◯			
Maxilla	◯			◯	◯	◯	◯		
Palatine	◯			◯	◯	◯	◯		
Dentary	◯			◯	◯				
Frontal	◯			◯		◯		◯	
Parietal		◯		◯				◯	
Squamosal	◯		◯				◯	◯	
Basiocciptial		◯	◯						◯
Nasal	◯			◯		◯			
Pterygoid	◯		◯		◯		◯		◯
Exoccipital		◯	◯					◯	◯
Basisphenoid		◯	◯				◯		◯
Lacrimal	◯			◯			◯	◯	
Alisphenoid		◯	◯				◯	◯	
Orbitosphenoid	◯		◯				◯	◯	
Petrosal		◯	◯						◯
Mastoid		◯	◯					◯	◯
Vomer	◯			◯	◯	◯			
Presphenoid	◯		◯						◯
Supraoccipital		◯	◯					◯	
Ectotympanic	◯		◯						◯
Goniale	◯		◯						◯

Developmental origin modules are derived from [62], ossification mode modules from [63], and phenotypic module from [34,64,65].

Integration of developmental timing was analyzed by testing for the conservation of rank orders within sets of traits across phylogeny [39]. If the order of development of a set of modular events is evolutionarily conserved, then the correlation of developmental sequence between taxa is expected to be high. This we examine statistically with the correlation coefficient for a given hypothesized events [39]. Ossification sequences in pairs of sister taxa were compared using Kendall's τ, which measures the degree of similarity of their sequences. Here, the correlation coefficient Kendall's τ is calculated as:

$$\text{T}=\frac{n_{\text{c}}-n_{\text{d}}}{\left(n_{\text{c}}+n_{\text{d}}+n_{\text{x}}\right){\left(n_{\text{c}}+n_{\text{d}}+n_{\text{y}}\right)}^{1∕2}}$$

in which *n*_c _is the number of concordant pairs of ranks, *n*_d _is the number of discordant pairs, *n*_x _is the number of tied events in the first taxon (that is, the number of simultaneous ossification events within the taxon), and *n*_y _is the number of tied events in the other taxon. We must note that the Kendall's τ correlation coefficient can be biased in cases of many tied events, but our dataset has enough resolution to calculate the coefficient.

The significance of the observed Kendall's τ between sister taxa was assessed for each module by comparing it within the null distribution of similar numbered sets of developmental events. We randomly sampled sets of a comparable number of developmental events from the whole events (iteration 1,000 times) and obtained the correlation value (that is, Kendall's τ) between taxa. For instance, the vault module contains nine bones. In this case, the rank correlation of nine vault bones between two sister taxa is first computed. Next, nine random events are sampled from the whole events, and then the correlation of nine randomly sampled events between the sister groups is computed. The null distribution for correlation values is obtained by conducting this procedure 1,000 times. If the vault module is significantly shifting as a whole, observed correlation value (that is, Kendall's τ) between the two taxa shall be ranked high among the null distribution of randomly obtained correlation values.

This test is simple if only two taxa are compared (that is, when comparing between terminal nodes), but comparisons on multiple species may involve statistical non-independence, which necessitates an approach that incorporates information on phylogeny [39]. In these cases, a version of Felsenstein's independent contrast [66] can circumvent the issue of non-independence of multiple species comparison [39]. First, the hypothetical ancestral developmental sequence is computed for each node. Then independent comparisons of sequences are conducted between pairs of independent nodes of the phylogeny [39]. Here the test statistic is the average Kendall's τ value for these node-pairs. The null distribution is obtained by calculating average correlation values of random samples (iteration 1,000 times). The sequence of ancestral nodes was reconstructed by averaging the sequences of sister taxa joined at a particular node [39]. The averaged sequence was considered as the hypothetical ancestral sequence and then used for a pairwise significance test of Kendall's τ. It should be noted that since the actual (extinct) ancestral sequence may not be simply the average of descendent taxa, this technique potentially produces biases. However, averaging is widely adopted in methods such as independent contrasts [9,66]. If heterochronies in ossification sequences have a strong modular pattern, such a pattern would be detectable with this approach [9,39]. In this study, a 0.05 significance level was adopted, and this significance level was corrected using Bonferroni threshold (*P *= 0.05/99). A random sampling procedure was done with Poptools (Pest Animal Control Co-operative Research Centre, Canberra, Australia) [67] and statistical analyses were conducted in PAST [68].

## Results

### Variation in ossification sequence

**Figure 5:**
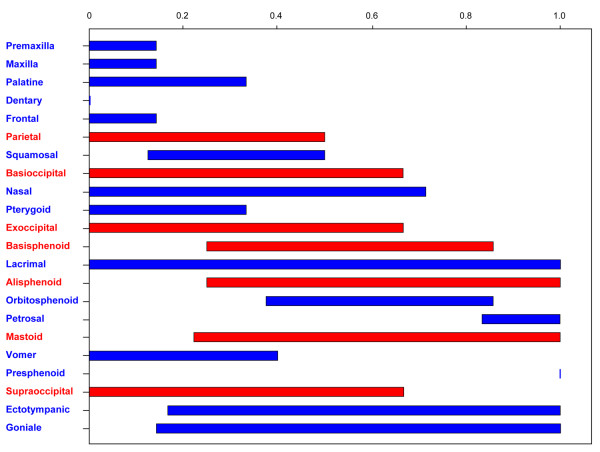
**Adjusted rank ranges of single bones across species**. Ranks range are from above 0 (ossifying first) to 1 (ossifying last). Dentary was always the first bone to ossify, and presphenoid was always the last bone to ossify in all species. Bones derived from the neural crest cells are colored in blue, and bones derived from the mesoderm are illustrated in red.

**Figure 6:**
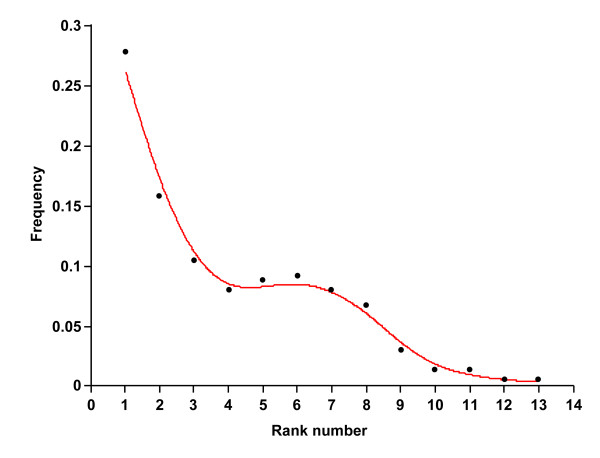
**Frequency variation plot of cranial ossifying events in this study**.

Ossification sequences of each species are listed in Table 2, and the frequency distribution of each stage is given in Additional file 1. The rank variation of each cranial element across lipotyphlan species is summarized in Figure 5. The distributions of the elements show that an ossification event occurs predominantly in the early stages of the whole ossification sequence (Figure 6). No intraspecific variation was found in ossification sequence for all species. The most interspecifically variable elements in ossification sequence were lacrimal, goniale, ectotympanic and mastoid. Presphenoid, dentary, frontal, maxilla, premaxilla and petrosal were among the least variable elements. The mean rank range for each phenotypic and developmental module is listed in Table 4. Among the five phenotypic modules, the vault module was the most variable followed by the basicranial and zygomatic modules. When developmental origin modules were compared, the mesoderm module was more variable than the neural crest cell module. Among the ossification mode modules, the cartilaginous bone module was more variable than the dermal bone module.

**Table 4:** Adjusted rank range for each bone set

		Rank range
Developmental origin	Neural crest cells	0.41
	Mesoderm	0.60
Ossification mode	Endochondral	0.55
	Dermal	0.38
Phenotypic module	Oral	0.23
	Nasal	0.31
	Zygomatic	0.50
	Vault	0.60
	Basicranial	0.55
Origin + ossification mode	Mesoderm (endochondral)	0.61
	Mesoderm (dermal)	0.50
	Neural crest cells (endochondral)	0.48
	Neural crest cells (dermal)	0.36

### Event paring and PARSIMOV analyses

A total of 231 event-pairs revealed 124 parsimony-informative characters (53.7% of total), with 67 characters constant (29.0%), and 40 variable pairs parsimony-uninformative (17.3%). Table 5 contains the consensus list of the movements in the timing of ossification of cranial elements detected by Parsimov. One potentially autapomorphic heterochronic pattern that characterizes lipotyphlans was found: the late onset of ossification of the lacrimal bone relative to the pterygoid. No heterochrony was found for nodes of Laurasiatheria, hedgehogs and shrews, respectively. On the other hand, moles were characterized by early ossifications of palatine (with respect to premaxilla and frontal), nasal (with respect to frontal and pterygoid), pterygoid (with respect to premaxilla, frontal and parietal), and vomer (with respect to premaxilla, palatine, frontal and parietal). The clade consisting of *Scapanus*, *Urotrichus*, *Mogera*, and *Talpa *was distinguished by a late onset of ossification of alisphenoid in relation to petrosal and presphenoid. The aquatic shrew *Ch. pltaycephala *was characterized by late shifts of maxilla and premaxilla (with respect to palatine, dentary and frontal), and lacrimal (with respect to basisphenoid and goniale) and early shifts of squamosal (with respect to palatine, dentary and frontal), nasal (with respect to palatine, dentary, frontal and pterygoid), pterygoid (with respect to palatine and frontal), exoccipital (with respect to palatine, frontal, parietal and supraoccipital), supraoccipital (with respect to frontal, parietal, nasal and pterygoid). The aquatic/fossorial mole *Co. cristata *was characterized by early development of basioccipital (with respect to premaxilla, maxilla, palatine, dentary, frontal and parietal, squamosal, pterygoid and supraoccipital), nasal (with respect to basiocciptial), exoccipital (with respect to premaxilla, maxilla, palatine, dentary, frontal, parietal, squamosal, basiocciptial, pterygoid and vomer), and supraoccipital (with respect to premaxilla, maxilla, palatine, dentary, frontal, parietal, pterygoid and vomer) and a late shift of lacrimal. The terrestrial mole *U. talpoides *was characterized by late shifts of nasal (with respect to frontal, pterygoid, exoccipital, basisphenoid, mastoid and supraoccipital), lacrimal (with respect to basisphenoid, alisphenoid, orbitosphenoid, petrosal, mastoid, presphenoid, ectotympanic and goniale), supraoccipital (with respect to squamosal, basiocciptial and basisphenoid), ectotympanic (with respect to basisphenoid, alisphenoid, orbitosphenoid, petrosal and presphenoid), and goniale (with respect to alisphenoid, orbitosphenoid, petrosal and presphenoid).

**Table 5:** List of the heterochronic movements reconstructed by the Parsimov method (consensus of ACCTRAN and DELTRAN)

Clade	event	movemet	...in relation to...
Laurasiatheria		No movement	
Chiroptera	Palatine	late	Parietal, Squamosal, Pterygoid, Ectotympanic
	Basioccipital	late	Nasal, Ectotympanic
	Exoccipital	late	Nasal, Alisphenoid
	Vomer	late	Nasal, Pterygoid, Ectotympanic
Rodentia	Premaxilla	late	Palatine, Squamosal
	Palatine	late	Parietal, Squamosal
	Exoccipital	early	Basiocciptial, Nasal, Pterygoid, Alisphenoid, Vomer
	Orbitosphenoid	late	Mastoid, Presphenoid
	Petrosal	early	Mastoid, Presphenoid
Lipotyphla	Lacrimal	late	Pterygoid
Erinaceidae		No movement	
*Erinaceus europaeus*	Frontal	early	Premaxilla, Maxilla, Dentary
	Parital	late	Premaxilla, Maxilla
	Pterygoid	early	Premaxilla, Maxilla, Dentary, Frontal, Vomer
*Erinaceus amurensis*		No movement	
Soricidae		No movement	
Soricinae		No movement	
*Cryptosis parva*	Parital	late	Premaxilla, Maxilla, Frontal
	Exoccipital	late	Squamosal, Nasal
	Vomer	early	Nasal, Pterygoid
*Chimarrogale platycephala*	Premaxilla	late	Palatine, Dentary, Frontal
	Maxilla	late	Palatine, Dentary, Frontal
	Squamosal	early	Palatine, Frontal, Parietal
	Nasal	early	Palatine, Frontal, Parietal, Pterygoid
	Pterygoid	early	Palatine, Frontal
	Exoccipital	early	Palatine, Frontal, Parietal, Supraoccipital
	Lacrimal	late	Basisphenoid, Goniale
	Supraoccipital	early	Frontal, Parietal, Nasal, Pterygoid
*Suncus murinus*	Parital	early	Palatine, Dentary, Frontal
	Basioccipital	early	Palatine, Squamosal
	Vomer	late	Pterygoid, Lacrimal
Talpidae	Palatine	early	Premaxilla, Frontal
	Nasal	early	Frontal, Pterygoid
	Pterygoid	early	Premaxilla, Frontal, Parietal
	Vomer	early	Premaxilla, Palatine, Frontal, Parietal
*Condylura cristata*	Basioccipital	early	Premaxilla, Maxilla, Palatine, Dentary, Frontal, Parietal,
			Squamosal, Pterygoid, Supraoccipital
	Nasal	early	Basiocciptial
	Exoccipital	early	Premaxilla, Maxilla, Palatine, Dentary, Frontal, Parietal,
			Squamosal, Basiocciptial, Pterygoid, Vomer
	Lacrimal	late	Basisphenoid, Ectotympanic, Goniale
	Supraoccipital	early	Premaxilla, Maxilla, Palatine, Dentary, Frontal, Parietal,
			Pterygoid, Vomer
*Scapanus + Urotrichus + Mogera + Talpa*	Alisphenoid	late	Petrosal, Presphenoid
*Scapanus orarius*	Basioccipital	late	Basisphenoid, Mastoid
	Lacrimal	early	Premaxilla, Maxilla, Palatine, Dentary, Frontal, Parietal
			Nasal, Pterygoid, Vomer
*Urotrichus + Mogera + Talpa*		No movement	
*Urotrichus talpoides*	Nasal	late	Frontal, Pterygoid, Exoccipital, Basisphenoid, Mastoid,
			Supraoccipital
	Lacrimal	late	Basisphenoid, Alisphenoid, Orbitosphenoid, Petrosal,
			Mastoid, Presphenoid, Ectotympanic, Goniale
	Supraoccipital	late	Squamosal, Basiocciptial, Basisphenoid
	Ectotympanic	late	Basisphenoid, Alisphenoid, Orbitosphenoid, Petrosal,
			Presphenoid
	Goniale	late	Alisphenoid, Orbitosphenoid, Petrosal, Presphenoid
*Mogera + Talpa*	Pterygoid	late	Premaxilla, Maxilla, Dentary, Parietal, Squamosal
*Mogera wogura*	Palatine	late	Premaxilla, Maxilla, Dentary, Parietal, Squamosal
	Frontal	late	Squamosal, Supraoccipital
	Orbitosphenoid	early	Basisphenoid, Ectotympanic
	Vomer	late	Premaxilla, Maxilla, Dentary, Parietal, Squamosal
	Goniale	early	Squamosal, Nasal, Pterygoid, Basisphenoid, Lacrimal,
			Supraoccipital, Ectotympanic
*Talpa*	Pterygoid	late	Palatine, Frontal
*Talpa europaea*	Frontal	early	Nasal
	Basioccipital	early	Squamosal
	Basisphenoid	early	Lacrimal, Mastoid
*Talpa occidentalis*	Frontal	late	Palatine, Vomer
	Squamosal	late	Nasal, Lacrimal, Mastoid
	Basioccipital	early	Premaxilla, Maxilla, Palatine, Dentary, Parietal, Vomer
	Pterygoid	late	Squamosal, Nasal, Lacrimal, Mastoid
	Exoccipital	early	Premaxilla, Maxilla, Palatine, Dentary, Parietal,
			Basiocciptial, Nasal, Vomer, Supraoccipital
	Supraoccipital	early	Premaxilla, Maxilla, Dentary, Parietal, Nasal

The results from the single ACCTRAN and DELTRAN analyses are also provided in Additional file 2. The separate results obtained from ACCTRAN and DELTRAN showed that Laurasiatheria is characterized by late shifts of premaxilla (with respect to parietal and squamosal), alisphenoid (with respect to nasal and basisphenoid), and orbitosphenoid (with respect to basisphenoid and petrosal). An early shift of parietal (with respect to maxilla and palatine) and late shifts of palatine (with respect to parietal and pterygoid), lacrimal (with respect to basioccipital, exoccipital, pterygoid and vomer), alisphenod (with respect to exoccipital and basisphenoid), presphenoid, ectotympanic and goniale were detected for Lipotyphla. The clade consisting of hedgehogs and shrews was characterized by early shifts of nasal (with respect to lacrimal and vomer) and supraoccipital (with respect to basioccipital, exoccipital and lacrimal) and a late shift of goniale (with respect to basioccipital and ectotympanic).

The consensus tree of two equally parsimonious trees resulting from the parsimony analysis of the event-pairing data (Additional file 3) was mostly incongruent with the phylogeny taken as reference (Figure 2). The terrestrial outgroup species *E. amurensis*, *S. murinus*, and *R. norvegicus *were clustered together. Although all six mole species were clustered together, this cluster was paraphyletic, given the position in it of *C. platycephala*'s position.

### Modularity analysis

Results of Poe's modularity test [39] are summarized in Table 6. No sets showed significant correlation for the hypothetical modules. For the analysis of phenotypic variational modules, one comparison showed a *P-*value of 0.027 (oral module in *T. europaea *vs. *T. occidentalis*), but this was not statistically significant under the Bonferroni threshold (0.05/99). Among the developmental origin modules, although three sister group comparisons (Erinaceidae-Soricidae node vs. Talpidae, Erinaceidae vs. Soricidae and *T. europaea *vs. *T. occidentalis*) showed *P-*values lower than 0.05 for the neural crest cell module, these values did not reach the Bonferroni corrected significance level. No significant correlation was found for the mesoderm module in any sister group comparison.

**Table 6:** Results of modularity analysis in pairs of studied taxa.

			Developmental origin module		Ossification mode module		Phenotypic module				
		
			Neural crest cells	Mesoderm	Endochondral	Dermal	Oral	Nasal	Zygomatic	Vault	Basicranial
*E. europaeus*	vs.	*E. amurensis*	0.877 (0.167)	0.843 (0.444)	0.825 (0.512)	0.608 (0.931)	0.667 (0.916)	0.783 (0.620)	0.754 (0.678)	0.814 (0.667)	0.867 (0.342)
*Cr.parva*	vs.	*Ch. platycephala*	0.803 (0.610)	0.904 (0.176)	0.888 (0.097)	0.613 (0.958)	0.405 (0.972)	-	0.737 (0.769)	0.787 (0.680)	0.866 (0.277)
*Cr.parva*+*Ch. platycephala*	vs.	*Su. murinus*	0.829 (0.310)	0.907 (0.166)	0.854 (0.250)	0.613 (0.941)	0.563 (0.978)	-	0.860 (0.454)	0.851 (0.472)	0.833 (0.494)
Erinaceidae	vs.	Soricidae	0.867 (0.043)	0.799 (0.639)	0.780 (0.708)	0.671 (0.944)	0.668 (0.917)	0.826 (0.550)	0.809 (0.620)	0.818 (0.500)	0.823 (0.486)
*T. europaea*	vs.	*T. occidentalis*	0.894 (0.041)	0.853 (0.333)	0.786 (0.597)	0.808 (0.487)	1.000 (0.027)	0.632 (0.981)	0.863 (0.110)	0.699 (0.875)	0.747 (0.750)
*Talpa*	vs.	*Mogera*	0.830 (0.148)	0.908 (0.111)	0.704 (0.903)	0.760 (0.666)	0.724 (0.708)	0.552 (0.982)	0.855 (0.269)	0.783 (0.625)	0.631 (0.944)
*Talpa*+*Mogera*	vs.	*Urotrichus*	0.732 (0.611)	0.852 (0.231)	0.608 (0.981)	0.716 (0.569)	*-*	0.546 (0.972)	0.771 (0.519)	0.805 (0.292)	0.562 (0.958)
*Talpa*+*Mogera*+*Urotrichus*	vs.	*Scapanus*	0.684 (0.630)	0.692 (0.657)	0.613 (0.875)	*-*	*-*	*-*	0.710 (0.611)	0.706 (0.486)	0.522 (0.917)
*Talpa*+*Mogera*+*Urotrichus*+*Scapanus*	vs.	*Cristatus*	0.731 (0.361)	0.753 (0.491)	0.722 (0.528)	*-*	*-*	*-*	0.797 (0.324)	0.700 (0.583)	0.630 (0.722)
Erinaceidae+Soricidae	vs.	Talpidae	0.839 (0.037)	0.764 (0.731)	0.730 (0.931)	0.711 (0.902)	*-*	*-*	0.799 (0.639)	0.695 (0.917)	0.795 (0.431)
Lipotyphla	vs.	*Myotis*	0.711 (0.370)	0.702 (0.602)	0.555 (0.944)	0.802 (0.111)	0.725 (0.403)	0.814 (0.278)	0.760 (0.380)	0.648 (0.667)	0.544 (0.875)

Kendall's τ is listed for each comparison and *P-*values are given in parentheses. Significance level was set as *P *< 0.05/99 (that is, *P *< 0.0005) by Bonferroni threshold. Values are not given for those sets in which Kendall's τ was indeterminate owing to low element numbers and/or high conservation of developmental sequence, denoted "-".

## Discussion

### Sequence heterochrony

It has recently been proposed that the sequence of cranial ossification is quite conservative and that few heterochronic shifts have occurred in mammalian evolution based on the study of 17 boreoeutherians [6]. This previous study, and other similar ones for other groups, used the fairly conservative approach of Parsimov [69] to detect heterochrony. To make our study comparable, we also used Parsimov. Although we confirmed the general vertebrate pattern (such as the early ossification of jaw bones with respect to the neurocranial bones [11,70]) in lipotyphlans, our analysis suggests that some heterochronies have occurred during the evolution of this group. For example, the late shifting of the lacrimal in respect to pterygoid characterizes Lipotyphla. In addition, moles exhibit considerably derived features, such as the accelerated development of the palatine, nasal, pterygoid and vomer. This indicates that the relatively early development of the vomeronasal complex is an autapomorphic feature of moles. In many mammals, bones in the vomeronasal region are known to ossify later than the anterior dermal bones (premaxilla, maxilla, palatine and frontal) [8,46,71]. However, it appears that the development of these bones occurs virtually simultaneously with the above mentioned anterior dermal bones in moles.

It is known that rostrum morphology is relatively robust and box-shaped in moles compared to that of shrews and hedgehogs. A robust snout is often used for boring through the soil and to push away encountered objects [72,73]. Mole snouts are equipped with highly touch-sensitive, domed mechanosensory organs called Eimer's organs, which are not found in other lipotyphlans, such as shrews, hedgehogs and solenodons [74,75]. Moles have poor eyesight and gather much of their information by probing the environment with the glabrous tip of their nose (rhinarium) [74,76]. Each Eimer's organ functions as a tactile receptor that contains a number of separate elements, including sensory receptors and supporting epidermal tissue in a specific configuration [74]. The drastic early shift of the vomeronasal complex may be related to the increased importance of the rostrum for fossorial lifestyle in moles, since the number of Eimer's organs reflects the surface area of the rhinarium [77], which is relatively large in moles.

In contrast, the shrew mole *U. talpoides*, which is more terrestrial [51,78], shows a secondary late shift of the nasal. This indicates that, whereas all fossorial moles develop the vomeronasal region early in embryogenesis, the terrestrial shrew-like *U. talpoides *is an exception. It is reported that *U. talpoides *possesses 1,310 Eimer's organs, which is a considerably small number compared to other true moles (*Mogera*, 2,200; *Talpa*, 2,200; *Scapanus*, 2,470; *Condylura*, 26,000; Catania 2,000). It is also noteworthy that relative size and robustness of the vomeronasal complex is reduced in *U. talpoides *compared to that of other moles and that its pointed gracile vomernasal organ resembles that of shrews [74,79,80]. In contrast to other fossorial moles, the more terrestrial *U. talpoides *has functional eyes and a pointed gracile rostrum used for sniffing as in shrews, whereas the role of the rostrum as a tactile sense and boring appendage is reduced [Hisashi Abe, personal communication]. It is conceivable that while the developmental timing of the vomeronasal complex shifted earlier in the common ancestor of moles, since the role of the snout was emphasized for subterranean lifestyle, the nasal reverted to a later development through secondary terrestrial adaptations in *U. talpoides*. This scenario results from the phylogenetic framework taken as reference, but it should be noted that the position of the shrew moles in talpid phylogeny is contested [47,51,52]

Another shift of the onset of ossification timing in *U. talpoides *is found for the supraoccipital. Although the supraoccipital has moved early in the common ancestor of moles, this bone reverts to a later development in *U. talpoides*. It is known that moles have undergone considerable morphological transformations associated with digging [81-83]. For example, the supraoccipital region is relatively increased in moles compared to other lipotyphlans [84] and provides the space to attach the enlarged *musculus rhomboideus *that functions as the major digging muscle [83]. Whereas the *m. rhomboideus capitis *is generally small in mammals, this muscle is considerably enlarged in moles [81,83]. Given that the enlarged supraoccipital provides the attachment site for the *m. rhomboideus capitis*, the early onset of the supraoccipital may be functionally coupled to the enlargement of this bone in moles. At the same time, the reduced occipital size in *U. talpoides *may reflect the reduced importance of digging muscles.

Another notable heterochrony found among lipotyphlans is that the lacrimal (with respect to basisphenoid and goniale), exoccipital (with respect to palatine, frontal and parietal), and supraoccipital (with respect to frontal and parietal) shift early in *Ch. platycephala *and in *Co. cristata*, respectively. These two phylogenetically distant species (Figure 2) are unique in comparison to other lipotyphlans studied here in having independently acquired a semi-aquatic lifestyle [85-87]. Although it is difficult to sort out the functional significance of early development of these bones, it may be related to adaptations for an aquatic lifestyle and swimming ability.

The phylogenetic relationships among shrews, hedgehogs and moles have been highly controversial [88-90]. While anatomical studies have conventionally suggested a shrew + mole clade to the excursion of hedgehogs [90], recent molecular studies supports the monophyly of shrews and hedgehogs [49,88,89]. Although event pairs are generally unsuitable for phylogenetic analysis due to the non-independence of data [6,11,91], it is worth noting that our exploratory parsimony based analysis resulted in a clustering of moles with the inclusion of *Ch. platycephala *(Additional file 3).

### Rank variability

Sánchez-Villagra *et al. *[6], whose study included 20 mammalian species, reported that the most interspecifically variable cranial elements in terms of relative ossification timing are the basioccipital, basisphenoid, jugal, parietal, pterygoid and squamosal. In contrast, the lacrimal, goniale, ectotympanic, mastoid, alisphenoid and nasal bones are found as the most variable elements during the onset of ossification in lipotyphlans (Figure 5). In a more inclusive study on Rodentia, it is reported that the parietal, alisphenoid, squamosal, jugal, pterygoid and basisphenoid are the most labile bones in terms of relative ossification timing [8]. Although it appears that alisphenoid is similarly variable in rodents and lipotyphlans, this bone is not reported to be highly variable across the major clades in mammals [6]. Presphenoid, dentary, frontal, premaxilla, maxilla and petrosal are the most conservative elements in lipotyphlans. Consistent with our results, petrosal, dentary and premaxilla are reported to be the least variable cranial elements in rodents [8]. Similarly, Sánchez-Villagra *et al. *[6] reported that the least variable elements include anterior facial bones, such as the dentary, maxilla, frontal and premaxilla. This suggests that facial elements, which generally ossify at early stages of bone development (Figure 5), are less variable in terms of ossification timing.

Overall, the amount of variability in timing of ossification onset in lipotyphlan cranial bones was as follows, in order of highest to lowest variability: mesoderm derived endochondral bones, mesoderm derived dermal bones, neural crest cell derived endochondral bones, and neural crest cell derived dermal bones (Table 4). Thus, the cartilaginous bones are more variable as a whole than dermal bones among the mesoderm derived bones, whereas endochondral bones are similarly more variable than dermal bones within the neural crest cell derived bones (Table 4). We also found that mesoderm derived bones tend to be more variable than neural crest cell derived bones. Sánchez-Villagra *et al. *[6] noted that the relative ossification timing of neurocranial traits, especially the basicranial bones, are more labile than the facial elements. The basicranial bones are also apparently highly variable as a whole within lipotyphlans (Figure 5, Table 4). However, the cranial base also includes less variable bones, such as presphenoid, petrosal and pterygoid. Instead, both developmental origin (mesoderm or neural crest cells) and ossification mode (dermal or endochondral) are coupled with the evolutionary lability of ossification timing.

### Modularity of ossification heterochrony

On the whole there was little integration across all the modules tested. No developmental module showed significant correlation for any sister group comparison. This indicates that lipotyphlan ossification heterochrony is not constrained by differences in developmental origins (mesoderm or neural crest cells) or ossification mode (dermal or endochondral). Although developmental origin and ossification mode of bones are considered to be highly influential to morphological evolution in vertebrates [62,92-94], it appears that ossification heterochrony is an exception to this pattern. It is possible that the independence of ossification timing of each bone may facilitate the labile alteration of ossification sequence and, thus, ease consequent evolutionary changes in phenotypes. In addition, the lack of clear integration in ossification heterochrony among phenotypic modules indicates that sequence heterochrony is virtually independent from morphometrically derived phenotypic modules of the cranium, which is consistent with the results reported by Goswami and colleagues [9,10]. Although adult phenotypic (metric) traits of the mammalian skull are reported to exhibit a highly modular pattern [for example, [33,34,95]], our results suggest that modularity in adult phenotypic traits is not strongly linked to integration in ossification sequence heterochrony. Functionally- or developmentally-integrated structures have often been suggested to exhibit coordinated shifts in developmental timing [10,11,13,96]. However, very few studies have explicitly examined the relationship between modularity and sequence heterochrony [10,95]. Our results presented here suggest that the hypothesis raised by Schoch [11] and others [12,13,96] - that there is a modular pattern in sequence heterochrony - is not supported for the case of lipotyphlans. Either the lipotyphlan case is an exception, or the sampling or methods used in previous analyses are in need of expansion or revision.

Although the integration of ossification heterochrony for *a priori *hypothetical modules was tested in this study, we did not attempt to detect heterochronic modules from our ossification dataset. We must admit that the modularity test and the following Bonferroni correction adopted in this study are rather conservative to detect modularity in sequence heterochrony. Development of analytical tools to detect modularity directly from sequence heterochrony data shall provide new avenues to understand the role of modularity in the evolution of vertebrate cranial diversity.

## Conclusion

The extensive examination of lipotyphlan craniogenesis reveals notable heterochronies in the onset of ossification. An early shift of developmental timing of the vomeronasal complex has occurred in the common ancestor of moles. This shift is most likely coupled with the robust nature of the rostrum for boring and tactile sensing in subterranean life. On the other hand, the nasal has reverted to a more typical later development in the more terrestrial shrew mole. This secondary shift in developmental timing can be linked to the secondary shift (concluded from phylogeny) to a terrestrial lifestyle and corresponding reduction of shrew mole digging and sensory requirements. Finally, we find no significant conservation of ossification timing within modules constructed depending on developmental origins or ossification modes. We reject the hypothesis that ossification sequence heterochrony is more likely to occur among different developmental modules. Although developmental origin and ossification mode of bones are considered to be highly influential to morphological evolution in vertebrates; in general, it is suggestible that lipotyphlan ossification heterochrony is not evolutionarily constrained by differences in developmental origins (mesoderm or neural crest cells) or ossification mode (dermal or endochondral).

## Competing interests

The authors declare that they have no competing interests.

## Authors' contributions

DBK and MRSV designed the study. DBK, HE, CM, SO, KK, MA and MRSV collected samples. DBK, GS and CPEZ conducted analysis of CT data. DBK analyzed the data and wrote the paper. All authors read, discussed and approved the final manuscript.

## Supplementary Material

Additional file 1 **Frequency distribution of specimens for each stage**. Ossification events are skewed toward earlier stages of sequences, suggesting a greater concentration of ossification events and/or less resolution early in the sequence. Click here for file

Additional file 2 **Heterochronies reported by the ACCTRAN and DELTRAN**. All the heterochronic events detected by the ACCTRAN and DELTRAN are summarized. Click here for file

Additional file 3 **Phenogram obtained from parsimony analysis of event-pair characters**. The consensus tree is obtained from parsimony analysis of event-paring scores. Although all six mole species were clustered together, the phenogram was mostly incongruent with the commonly accepted phylogenetic relationships. Click here for file
