# Supplementary figures and images for: Heterochrony and developmental modularity of cranial osteogenesis in lipotyphlan mammals
Source: EvoDevo. 2011 Nov 1;2:21. doi: 10.1186/2041-9139-2-21 (PMC3247175; doi:10.1186/2041-9139-2-21)

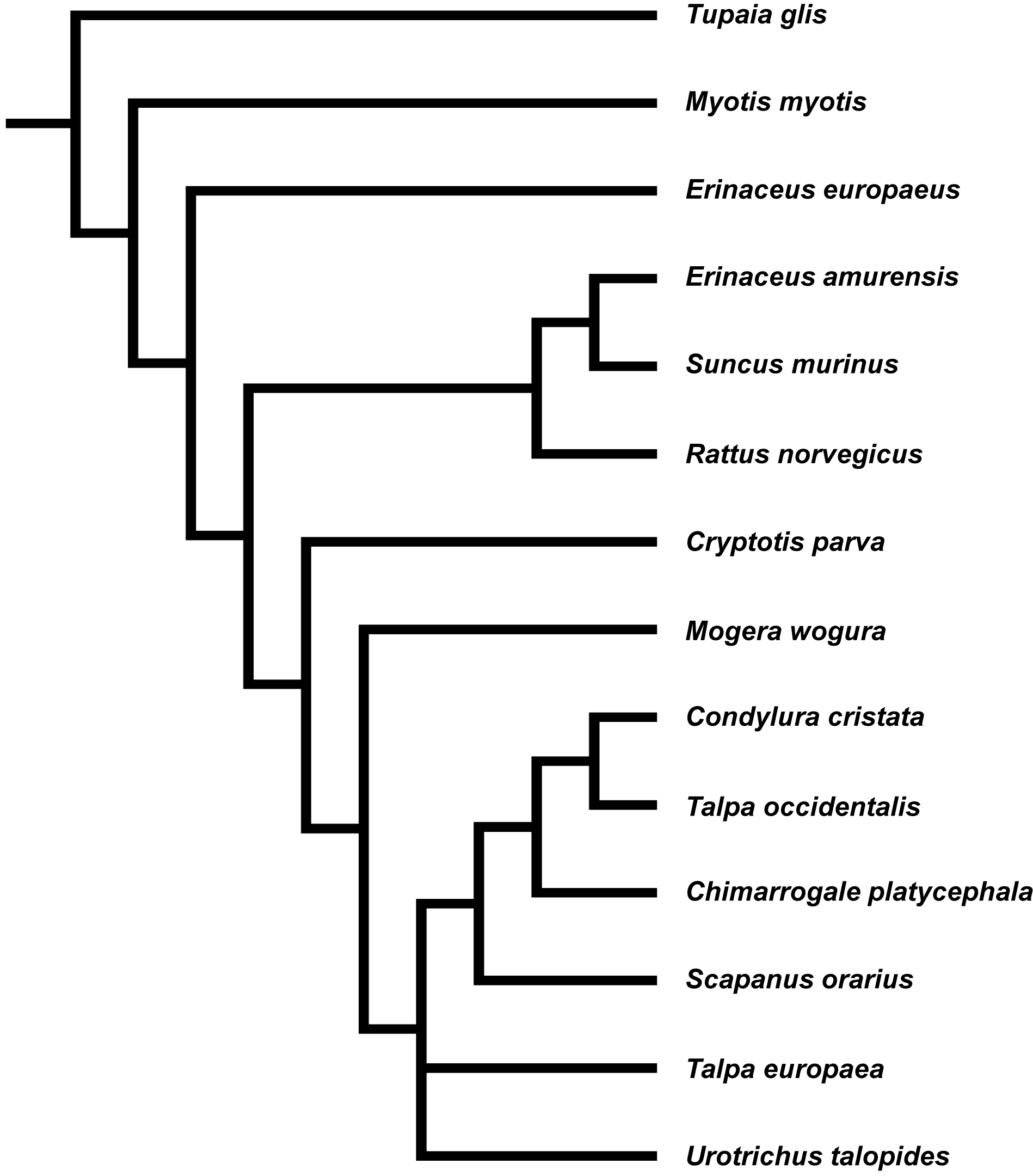

Supplement: Additional file 3 — Phenogram obtained from parsimony analysis of event-pair characters. The consensus tree is obtained from parsimony analysis of event-paring scores. Although all six mole species were clustered together, the phenogram was mostly incongruent with the commonly accepted phylogenetic relationships. [file 2041-9139-2-21-S3.TIFF]
